# A comparison of coronavirus disease 2019 and seasonal influenza surveillance in five European countries: France, Germany, Italy, Spain and the United Kingdom

**DOI:** 10.1111/irv.12941

**Published:** 2021-12-05

**Authors:** Thierry Rigoine de Fougerolles, Joan Puig‐Barbera, George Kassianos, Philippe Vanhems, Jorg Schelling, Pascal Crepey, Raul Ortiz de Lejarazu, Filippo Ansaldi, Markus Fruhwein, Cristina Galli, Anne Mosnier, Elena Pariani, Anvar Rasuli, Olivier Vitoux, John Watkins, Thomas Weinke, Hélène Bricout

**Affiliations:** ^1^ CVA, Healthcare Practice Paris France; ^2^ Vaccines Research Fisabio Valencia Spain; ^3^ Royal College of General Practitioners, UK and British Global & Travel Health Association London UK; ^4^ Department of Hygiene and Epidemiology, Public Health, Epidemiology and Evolutionary Ecology of Infectious Diseases (PHE^3^ID) – Inserm ‐ U1111 – UCBL Lyon University Hospital and Centre International de Recherche en Infectiologie (CIRI) Lyon France; ^5^ Medical Faculty Ludwig‐Maximilians‐University Munich Germany; ^6^ School of Advanced Studies in Public Health University of Rennes Rennes France; ^7^ Valladolid NIC University of Valladolid Valladolid Spain; ^8^ Department of Health Sciences University of Genoa Genoa Italy; ^9^ Dr. Fruehwein & Partners, Practise for General Medicine, Travel Medicine and Tropical Diseases Munich Germany; ^10^ Department of Biomedical Sciences for Health University of Milan Milan Italy; ^11^ Réseau des GROG Open Rome Paris France; ^12^ Medical Department Sanofi Pasteur Lyon France; ^13^ Division of Population Medicine, School of Medicine Cardiff University Cardiff UK; ^14^ Klinikum Ernst von Bergmann Medizinische Klinik, Gastroenterologie, Infektiologie, Pneumologie Potsdam Germany

**Keywords:** burden, COVID‐19, epidemiology, Europe, influenza, surveillance

## Abstract

**Background:**

In response to the coronavirus disease (COVID‐19) outbreak that unfolded across Europe in 2020, the World Health Organisation (WHO) called for repurposing existing influenza surveillance systems to monitor COVID‐19. This analysis aimed to compare descriptively the extent to which influenza surveillance systems were adapted and enhanced and how COVID‐19 surveillance could ultimately benefit or disrupt routine influenza surveillance.

**Methods:**

We used a previously developed framework in France, Germany, Italy, Spain and the United Kingdom to describe COVID‐19 surveillance and its impact on influenza surveillance. The framework divides surveillance systems into seven subsystems and 20 comparable outcomes of interest and uses five evaluation criteria based on WHO guidance. Information on influenza and COVID‐19 surveillance systems were collected from publicly available resources shared by European and national public health agencies.

**Results:**

Overall, non‐medically attended, virological, primary care and mortality surveillance were adapted in most countries to monitor COVID‐19, although community, outbreak and hospital surveillance were reinforced in all countries. Data granularity improved, with more detailed demographic and medical information recorded. A shift to systematic notification for cases and deaths enhanced both geographic and population representativeness, although the sampling strategy benefited from the roll out of widespread molecular testing. Data communication was greatly enhanced, contributing to improved public awareness.

**Conclusions:**

Well‐established influenza surveillance systems are a key component of pandemic preparedness, and their upgrade allowed European countries to respond to the COVID‐19 pandemic. However, uncertainties remain on how both influenza and COVID‐19 surveillance can be jointly and durably implemented.

## 1 INTRODUCTION

The first cases of coronavirus disease (COVID‐19), caused by severe acute respiratory syndrome coronavirus 2 (SARS‐CoV‐2), were reported in Europe in January 2020.^1^ On 11 March 2020, the World Health Organisation (WHO) identified COVID‐19 as a pandemic.^2^ A multicomponent surveillance system is critical to monitor the epidemiology, virology and health consequences of this new respiratory pathogen but is compounded by the novelty of the virus, the definition of the symptomatology and the role of asymptomatic carriage.^3^ The WHO called for a repurposing of influenza surveillance systems as an efficient, cost‐effective and sustainable approach to respond quickly to COVID‐19^4^, ^5^ and the European Centre for Disease Prevention and Control (ECDC) subsequently issued guidance on surveillance strategies for COVID‐19.^6^

Influenza surveillance systems are well established in Europe, reflecting the WHO Global Influenza Surveillance and Response System requirements^7^ and supplemented by national tools or pan‐European initiatives such as InfluenzaNet for non‐medically attended participatory syndromic surveillance systems^8^ and EuroMOMO for excess mortality modelling.^9^ However, the objectives of routine seasonal influenza surveillance and COVID‐19 surveillance are not exactly the same. For both influenza and COVID‐19, surveillance enables an understanding of the epidemiology, virology and geographic spread of disease and measuring of disease burden, severity and impact of prevention measures. However, in the context of a pandemic, COVID‐19 surveillance must also take into account disease containment using a test, track and trace approach and assess the overall disruption caused to healthcare systems, economies and communities.^10^ Additionally, the higher transmission rate for SARS‐CoV‐2 means that its surveillance systems may need to be more reactive.

Although the enhancement and expansion of existing influenza systems for COVID‐19 surveillance could benefit influenza surveillance, there could also be a diversion of resources from influenza surveillance towards COVID‐19. A solution could be to combine surveillance, without which a shift in favour of COVID‐19 could have an impact on the quality of influenza surveillance data.^11^ The public health consequences could include an impact on the selection of seasonal influenza vaccine strains, less accurate burden of influenza disease estimates that could lead to reduced public awareness of disease severity, increased vaccine hesitancy, reduced vaccine coverage and reduced population‐level protection if influenza viruses were to circulate as in previous years. Furthermore, WHO has warned that a shift from influenza to COVID‐19 surveillance may make it more difficult to avert future influenza pandemics.^12^

The purpose of this research was to understand to what extent influenza surveillance systems in five western European countries were repurposed, strengthened or complemented by new components for COVID‐19 surveillance and the implications for future influenza surveillance.

## 2 METHODS

### 2.1 Comparative framework

We applied a pre‐existing framework of influenza surveillance systems in France, Germany, Italy, Spain and the United Kingdom with seven subsystems (Table 1) and five evaluation criteria (Table 2) to COVID‐19 surveillance.^1^, ^13^

The seven subsystems—(1) non‐medically attended surveillance, (2) virological surveillance, (3) community surveillance, (4) outbreak surveillance, (5) primary care surveillance, (6) hospital surveillance, and (7) mortality surveillance—encompass a list of 20 comparable outcomes following a scale of severity from non‐medically attended suspected infection to lethal cases (Table 1). The five evaluation criteria—granularity, timing, representativeness, data sampling, and communication—are associated with a list of subcriteria defined in WHO guidance (Table 2). The framework was updated to include an extra outcome in the Community Surveillance subsystem (serum samples for population immunity estimates: Outcome 3.2 in Table 1) and two extra subcriteria in the Communication evaluation criterion (use of infographics/dashboard and interactive map: Table 2). Data in the United Kingdom are presented separately for England, Wales, Scotland and Northern Ireland.

**TABLE 1:** Influenza surveillance subsystems and comparative outcomes

Surveillance subsystem	Outcome
1. Non‐medically attended community surveillance	1.1. ARI/ILI/COVID‐like cases and/or incidence rates
	1.2. Proportion of ARI/ILI/COVID‐like cases seeking care
2. Virological surveillance	2.1. ARI/ILI/COVID‐like specimens for virus typing & subtyping
	2.2. ARI/ILI/COVID‐like specimens for virus genome sequencing
	2.3. ARI/ILI/COVID‐like specimens for antiviral drug resistance
3. Community surveillance	3.1. Notified biologically/laboratory‐confirmed cases
	3.2. Serum samples for population immunity estimates
4. Outbreak surveillance	4.1. ARI/ILI/COVID‐like outbreaks in closed settings
	4.2. Biologically/laboratory‐confirmed outbreaks in closed settings
5. Primary care surveillance	5.1. ARI/ILI/COVID‐like GP visits and/or incidence rates
	5.2. Biologically/laboratory‐confirmed GP visits and/or incidence rates
	5.3. Associated excess GP visits
	5.4. Associated excess work loss cases
6. Hospital surveillance	6.1. ILI/COVID‐like or biologically/laboratory‐confirmed Emergency Department visits
	6.2. SARI/ILI/COVID‐like hospital admissions
	6.3. Biologically/laboratory‐confirmed hospital admissions
	6.4. Associated excess hospital admissions
	6.5. Biologically/laboratory‐confirmed ICU admissions
7. Mortality surveillance	7.1. Diagnosed or biologically/laboratory‐confirmed deaths
	7.2. Associated excess deaths

*Note*. Adapted from El Guerche‐Séblain et al.^13^

Abbreviations: GP, general practitioner; ICU, intensive care unit; ILI, influenza‐like illness; (S)ARI, (severe) acute respiratory illness.

^a^ Not included in El Guerche‐Séblain et al.^13^

**TABLE 2:** Surveillance evolution criteria and associated subcriteria (primary care surveillance subsystem example)

Criteria	Sub‐riteria	WHO guidance^a^
Granularity	Age group	Recommended as a minimum: 0–1, 2–4, 5–14, 15–49, 50–64, 65 + years and ideally additional age strata for under 2 years including 0 to <6 months, 6 month to <1 year, 1 to <2 years
	Gender	Where possible data should be extracted by gender
	Risk condition	Recommended as a minimum: pregnancy status & presence of chronic pre‐existing medical illness (es): chronic respiratory disease, asthma, diabetes, chronic cardiac disease, chronic neurological or neuromuscular disease, haematological disorders, immunodeficiency (including Human Immunodeficiency Virus)
	Location	Considered as essential, especially for burden estimation for a given area based on data from sentinel sites
	Virology	Types and subtypes of viruses detected during the week
	Severity	Additional data to consider: signs and symptoms of illness & patient outcome (death, survival)
	Treatment	Exposure to influenza antiviral drugs during the last 14 days? If yes, name of antiviral
	Vaccination status	Additional data to consider: Seasonal influenza vaccination status and date of administration
Timing	Frequency	Epidemiological and virological data collected from the sentinel sites should be reported to the national health authorities on a weekly basis
	Time period	In temperate climate zones where influenza seasonality is well understood, data collection and reporting should occur at a minimum during the known influenza season and for a short period preceding and following the season
Representativeness	Geographical representativeness	National ‐ sentinel sites should include patients that will appropriately represent the population
	Population representativeness	The population served by the sentinel site should be representative of the target age and socioeconomic groups in the population under surveillance
	Number of settings	There is no ideal number of sentinel sites in a country. Start small with one or a few sentinel sites and only expand if these function well. Minimal information that should be presented in the weekly report includes number of sentinel sites reporting
	Proportion of facilities	Ideally the following analyses can be presented in an annual report: data from the monitoring of the system: proportion of sentinel sites reporting weekly to the national level; and if feasible, the proportion of sentinel sites regularly submitting specimens for laboratory testing
Sampling Strategy	Surveillance type	Sentinel surveillance
	ARI/ILI definition	An acute respiratory infection with fever ≥38 °C and cough with onset within the last 10 days
	Sampling	A systematic approach to case selection that does not leave the choice of cases to test or gather data from up to healthcare providers (other than to determine that the case meets the definition), and that covers different times of the day and different days of the week is likely to be the most pragmatic, whilst providing reasonably representative data
	Test type	Reverse transcriptase‐polymerase chain reaction (RT‐PCR) is the most sensitive method for detecting influenza virus and is the recommended influenza surveillance assay for laboratories
Communication	In annual report	Yearly surveillance report with surveillance and risk factor data should be produced
	In weekly report	Weekly surveillance reports should be produced and made accessible to relevant partners
	Delay in release	Reports should provide timely information on influenza activity and types of influenza viruses circulating
	Data can be extracted	Whenever feasible, such reports should be available to the public on the national surveillance website
	Infographics/dashboard^b^	Availability of user‐friendly infographics and/or dashboard
	Interactive map^b^	Availability of a user‐friendly interactive map

*Note*. Adapted from El Guerche‐Séblain et al.^13^ Further information is included in the Supporting Information.

Abbreviations: ARI, acute respiratory illness; ILI, influenza‐like illness; RT‐PCR, reverse transcriptase‐polymerase chain reaction.

^a^ From WHO global epidemiological surveillance standards for influenza^49^ and WHO manual for estimating disease burden associated with seasonal influenza.^39^

^b^ Not included in El Guerche‐Séblain et al.^13^

### 2.2 Data collection

Information to describe influenza and COVID‐19 surveillance systems were extracted from publicly available sources during the first wave in Europe, using online ECDC and WHO resources followed by national public health resources in each country, in particular: Santé publique (SpF) (France), Robert Koch Institute (RKI) (Germany), Istituto Superiore di Sanità (ISS) (Italy), Instituto de Salud Carlos III (ISCIII) (Spain) and Public Health England (PHE)/Public Health Wales/Public Health Scotland/Public Health Northern Ireland (UK).^14^, ^15^, ^16^, ^17^, ^18^, ^19^, ^20^, ^21^, ^22^, ^23^, ^24^, ^25^, ^26^, ^27^, ^28^ Influenza information was from weekly and annual reports, and COVID‐19 information was from daily and weekly reports. Interactive dashboards and maps as well as description of COVID‐19 surveillance system were used in a complementary manner when available.^29^, ^30^, ^31^, ^32^ Further details were sourced from the technical guidelines on case definition, data reporting or outbreak management when available.

### 2.3 Data interpretation

The results of the comparative analysis were reviewed, discussed and adjusted with a panel of 28 experts (including the authors), incorporating epidemiologists, virologists, general practitioners, public health researchers and pharmaceutical industry medical experts from each country during a virtual event in September 2020.

## 3 RESULTS

### 3.1 Surveillance subsystems

Existing influenza surveillance systems were to a large extent also used for COVID‐19 surveillance in each country, but were expanded, and complemented with additional surveillance components. Table 3 (France, Germany, Italy, Spain) and Table 4 (UK nations) highlight the additional components implemented for COVID‐19 surveillance for the seven subsystems, and the detail for each subsystem is included in the Supporting Information.

**TABLE 3:**
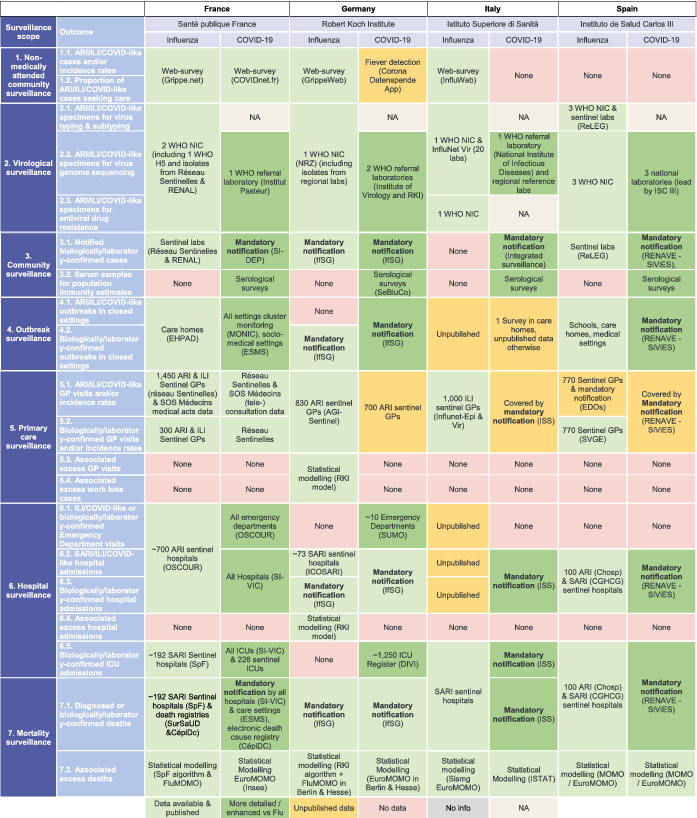
Overview of comparison of influenza and COVID‐19 surveillance systems in France, Germany, Italy and Spain

Abbreviations: AGI, Arbeitsgemeinschaft für Influenza (influenza working group); ARI, acute respiratory illness; CépiDc, Centre d'épidémiologie sur les causes médicales de décès (epidemiological centr on medical causes of deaths); CGHCG, Casos graves hospitalizados confirmados de gripe (severe hospitalised influenza cases); DIVI, Interdisziplinären Vereinigung für Intensiv‐ und Notfallmedizin; EDO, Enfermedades de Declaración Obligatoria (disease under mandatory notification); EHPAD, Etablissement d'Hébergement pour Personnes Âgées Dépendantes (nursing home); ESMS, Surveillance établissements sociaux et médico sociaux; GP, general practitioner; ICOSARI, Hospital surveillance system for severe acute respiratory infections; ICU, intensive care unit; IfSG, Infektionsschutzgesetz (law on protection against infectious diseases); ILI, influenza‐like illness; ISC, Instituto de Salud Carlos; ISS, Istuto Superiore di Sanità; ISTAT, Istituto Nazionale di Statistica; MOMO, Mortality Monitoring; MONIC, Monitoring of Clusters; NA, Not Applicable; NIC, National Influenza Centre; OSCOUR, Organisation de la Surveillance COordonnée des Urgences (organisation for coordinated emergency department surveillance); ReLEG, Red de Laboratorios de Gripe en España; RENAL, Réseau national des laboratoires hospitaliers; RENAVE, Red Nacional de Vigilancia Epidemiológica; RKI, Robert Koch Institute; SI‐DEP, Système d'information national de dépistage; SI‐VIC, Système d'information pour le suivi des victimes d'attentats et de situations sanitaires exceptionnelles (system for the monitoring of casualties from terror attacks and exceptional medical events); SIVIES, Sistema para la vigilancia en España; SurSaUD, Surveillance Sanitaire des Urgences et des Décès (public health surveillance of emergencies and deaths); SVGE, Sistema Centinela de Vigilancia de Gripe en España (Spanish influenza surveillance sentinel system); WHO, World Health Organisation.

**TABLE 4:**
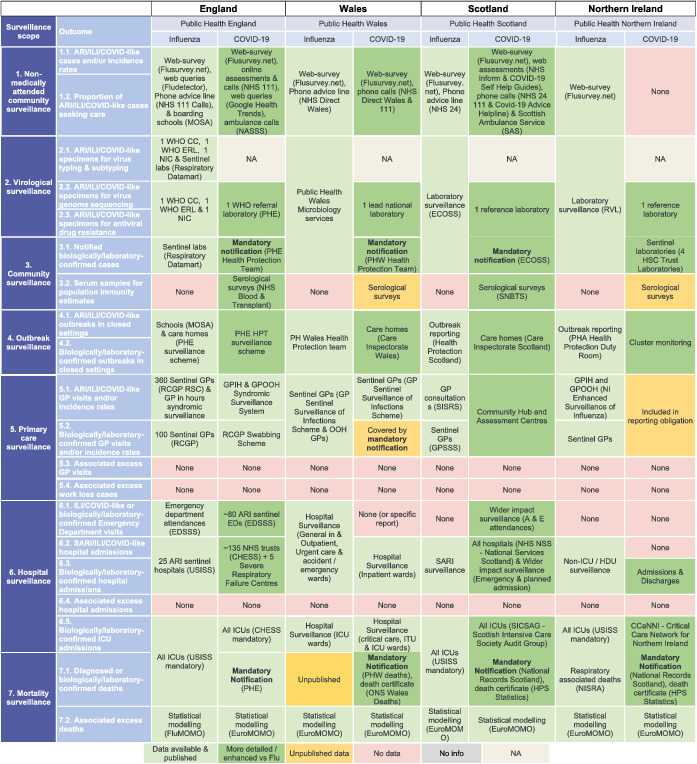
Overview of comparison of influenza and COVID‐19 surveillance systems in the United Kingdom

Abbreviations: CCaNNI, Critical Care Network for Northern Ireland; CC, Collaborating Centre; CHESSS, COVID‐19 Hospitalisation in England Surveillance System; ECOSS, Electronic Communication of Surveillance in Scotland; EDSSS, Emergency Department Syndromic Surveillance System; GPIH, General Practitioner in‐hours; GPOOH, General Practitioner out‐of‐hours; GPSSS, GP sentinel swabbing scheme; HDU, High Dependency Unit; HPS, Health protection Scotland; HSC, Health Security Committee; ICU, intensive care unit; MOMO, Mortality Monitoring; MOSA, Medical Officers of Schools Association; NA, Not Applicable; NASSS, National Ambulance Syndromic Surveillance System; NHS, National Health Service; NIC, National Influenza Centre; NISRA, Northern Ireland Statistics and Research Agency; ONS, Office for National Statistics; PHA, Public Health Agency; PHE, Public Health England; PHW, Public Health Wales; RCGP, Royal College of General Practitioners; RSC, Research and Surveillance Centre; SAS, Scottish Ambulance Service; SICSAG, Scottish Intensive Care Society Audit Group; SNBTS, Scottish National Blood Transfusion Service; USISS, UK Severe Influenza Surveillance System; WHO, World Health Organisation.

#### 3.1.1 Non‐medically attended surveillance

France and the United Kingdom repurposed voluntary web surveys to study the prevalence of COVID‐19 symptoms, as well as to monitor changes in healthcare‐seeking behaviours.^33^, ^34^ In the United Kingdom, National Health Service web assessments and calls and web queries (Google Health Trends) were repurposed, whilst monitoring of ambulance calls was added to surveillance in England with the National Ambulance Syndromic Surveillance System (NASSS) and Scotland with the Scottish Ambulance Service (SAS). Germany, Italy and Spain did not repurpose their web surveys nor implement new components for COVID‐19.

#### 3.1.2 Virological surveillance

Virological surveillance was repurposed and adapted, as national influenza centres refocused virus characterisation and genome sequencing capabilities to provide the WHO and the Global Initiative on Sharing All Influenza Data (GISAID) platform with SARS‐CoV‐2 virological data.^35^, ^36^

#### 3.1.3 Community surveillance

A mandatory notification system of all tested and laboratory‐confirmed COVID‐19 cases was implemented in each country, which had previously only been used in Germany for seasonal influenza.

In France, there was a shift from sentinel and national networks to an extensive surveillance scheme covering >5,500 sites and providing daily aggregated data. Germany implemented widespread testing from the start of the pandemic, mobilising >20,476 primary care practices, 959 hospitals and 70 laboratories, and in some regions, testing was free even for those without symptoms. In Italy and Spain, it is also assumed that all public medical settings and laboratories have been mobilised, although insights on completeness of data were not available. In the United Kingdom, following a massive roll‐out of reverse transcriptase polymerase chain reaction (RT‐PCR) testing, health authorities also mandated the reporting of all tested and laboratory‐confirmed patients in medical and non‐medical settings; these data were notified by a network of laboratories to the relevant national public health agencies.

To complement RT‐PCR molecular testing, serological testing was introduced in each country to measure the proportion of the population having been infected and developed antibodies.

#### 3.1.4 Outbreak surveillance

Influenza outbreak monitoring was repurposed for COVID‐19 monitoring and significantly enhanced in each country supported by guidance from the ECDC and following considerable COVID‐19 mortality and morbidity reported at care homes, which accounted for 21% of fatal cases in England, 37% in Germany and 66% in Spain.^37^ In France, where there is a system for notification of nosocomial infections, outbreak monitoring was expanded from care homes, which were the only closed setting included for influenza surveillance, to all medical and socio‐medical settings, and complemented by cluster monitoring in the community. Similarly, COVID‐19 outbreaks in Germany were notified for a wider range of closed settings than for influenza, in particular private and non‐medical settings. In Italy and Spain, regional health authorities also monitored COVID‐19 outbreaks in closed settings, such as care homes and schools, in the context of their decentralised healthcare system. In the United Kingdom, influenza outbreak surveillance was repurposed to monitor acute respiratory infection (ARI) outbreaks in a wider range of closed settings, and to investigate COVID‐19 aetiology.

#### 3.1.5 Primary care surveillance

Sentinel GP schemes were rapidly repurposed or shifted to universal and mandatory notification to provide samples to regional/national reference laboratories for detection of SARS‐CoV‐2. France also repurposed a network monitoring (tele)consultations, and England repurposed its syndromic surveillance to report clinically diagnosed cases on a weekly basis.

#### 3.1.6 Hospital surveillance

Secondary care surveillance was vastly repurposed and expanded to allow the monitoring of COVID‐19 severe outcomes and to assess the pressure on the secondary care system.

In France, existing systems were repurposed, and new systems were implemented to monitor patients with suspected or confirmed COVID‐19 infection in outpatient and inpatient wards on a daily basis. For example, 226 sentinel intensive care unit (ICU) departments that previously reported severe influenza cases became dedicated to the description of severe COVID‐19 cases, whilst the SIVIC system (Système d'Identification Unique des Victimes) was introduced to report COVID‐19 patients across all hospital wards. The higher burden of disease for COVID‐19 compared with influenza meant that the organisation of care was changed and so the capacity to count cases was not similar for the two situations. In Germany, similar to influenza surveillance, medical personnel were mandated to report hospitalised patients for COVID‐19, based on clinical or laboratory diagnosis. Additionally, COVID‐19 hospital surveillance was complemented by real‐time monitoring of emergency department attendance, and a register to record COVID‐19 ICU admissions and mechanical ventilation was expanded from 50 to about 1,300 ICU departments. Italian hospital surveillance for influenza was upgraded in response to COVID‐19 to provide mandatory data on both COVID‐19 hospital and ICU admissions. In Spain, two sentinel hospital systems were used to monitor hospitalised influenza cases and severe hospitalised cases with data on ICU transfer; this system shifted to mandatory notification providing the daily number of COVID‐19 hospital and ICU admissions. In England, existing sentinel schemes for influenza surveillance were repurposed and expanded, and daily COVID‐19 data on patients admitted to ICU and under invasive ventilation are provided.

#### 3.1.7 Mortality surveillance

Tools to monitor influenza mortality were repurposed and enhanced for COVID‐19 surveillance, with the number of suspected and/or laboratory‐confirmed COVID‐19 deaths reported daily through mandatory notification systems.

COVID‐19 deaths in France were notified for hospitals and social/medical settings, and data from electronic death certificates were used to estimate COVID‐19 attributable mortality (covering 25% of all deaths, compared to 20% for the previous influenza season). In Germany, systematic reporting of clinically diagnosed or laboratory‐confirmed deaths for COVID‐19 was provided by type of setting. In Italy and Spain, national public health agencies collected all clinically diagnosed or laboratory‐confirmed COVID‐19 deaths on a daily basis, mostly in hospitals, but deaths occurring in Spanish nursing homes were not included in the first wave of the pandemic due to limited laboratory testing capacity. Additionally, Italy used a rapid mortality surveillance system in 19 major Italian cities to produce estimates of overall excess mortality. In England, two new metrics were introduced for COVID‐19‐related deaths, counting deaths within ≤28 and <60 days of a laboratory‐confirmed positive test or having COVID‐19 on the death certificate. In August 2020, PHE aligned with the other UK nations counting a COVID‐19‐related death as one within 28 days of a laboratory‐confirmed positive COVID‐19 test.

Additionally, EuroMOMO^9^ was repurposed to approximate the overall mortality attributable to COVID‐19 through all‐causes excess mortality in each country. No estimate of the excess mortality specifically attributable to COVID‐19 has been done, whereas the FluMOMO model^38^ for influenza‐specific attributable mortality is run annually in France, England and parts of Germany.

### 3.2 Surveillance criteria

The characteristics of COVID‐19 surveillance across the five evaluation criteria have significantly evolved in each country compared with influenza surveillance. Data were made available in a more granular manner per age group, sex or location, as well as more frequently, better representing the national situation through improved coverage and systematic testing strategies. A step change was made in the sampling strategy given the massive use of RT‐PCR testing, and to a lesser extent genome sequencing, and there were major improvements in data communication, making the data more accessible and user‐friendly for the lay public.

#### 3.2.1 Data granularity

As for influenza surveillance, national statistics were published with data stratified by age, sex, presence of risk condition and location. For COVID‐19 surveillance, each country adopted an age stratification suggested by the WHO for influenza (0 to <2 years, 2 to <5 years, 5 to <15 years, 15 to <50 years, 50 to <65 years, ≥65 years) and often further stratified data per 10‐year age group.^39^ Moreover, the availability of data per underlying condition, especially for hospital and ICU admissions as well as deaths, and particularly in Italy and Spain, represented major improvements. Complementing national statistics, laboratory‐confirmed cases and deaths were often provided by region, and even county, allowing visualisation of disparities within a territory. In Germany and Spain, in contrast to influenza surveillance, the type of setting of COVID‐19 exposure was also available. Additionally, in England, COVID‐19 data are provided by ethnicity, and in Northern Ireland, coronavirus‐related health inequalities are available.

Beyond demographic information, importance was put on COVID‐19 symptoms and complications. Contrary to influenza surveillance, most countries provided a breakdown between asymptomatic and symptomatic cases. The level of severity of the infection was also closely monitored, and data on hospitalisation or mechanical ventilation were often available. The outcome of a hospital admission, that is, death or recovery, was also part of the daily national statistics, and in Italy, cases were classified using a severity scale.

#### 3.2.2 Data timing

Surveillance frequency was adapted to improve responsiveness, with daily reporting of COVID‐19 data compared with weekly reporting for influenza. COVID‐19 surveillance also shifted from a seasonal to a year‐round approach.

#### 3.2.3 Data representativeness

There was a significant shift from sentinel‐based influenza surveillance in most countries, that relied on volunteer medical personnel in primary and secondary care, to a mandatory notification system, encompassing all medical settings as was already performed for influenza surveillance in Germany. Syndromic surveillance based on ARI and influenza‐like (ILI) symptoms was also replaced by a systematic surveillance of possible COVID‐19 cases, regardless of the presence of the symptoms, but taking into account travel history and person‐to‐person contacts.

The systematic surveillance enforced through mandatory notification was intended to cover all medical settings, with more hospitals and laboratories involved in surveillance of COVID‐19 than influenza. Nursing/care homes also became better covered, and the aetiology of any outbreak was systematically investigated through testing. Lastly, surveillance of COVID‐19 in closed settings, unlike influenza, was not limited to state‐run institutions such as educational facilities or prisons but expanded to include private settings such as workplaces, restaurants and cafes.

#### 3.2.4 Sampling strategy

The massive and unprecedented use of RT‐PCR testing led to a step change in determining the viral aetiology of suspected cases with or without symptoms, and laboratory‐confirmed infection became the gold standard to accurately record COVID‐19 cases, complemented by results from serological surveys. However, the five countries evaluated did not use a harmonised clinical definition for possible, probable or confirmed cases, or deaths, and relied mainly on the observation of clinical symptoms.

#### 3.2.5 Data communication

For seasonal influenza, virology and epidemiology data are communicated through technical weekly and annual reports. For COVID‐19, public health and governmental agencies developed user‐friendly interfaces, with interactive dashboards and maps, additional to the publication of technical reports. The use of these communication tools, combined with regular interventions from public health officials and subject matter experts, relayed by the mass and social media, contributed to enhanced disease awareness. Access to COVID‐19 data was considerably improved compared with influenza, with many countries allowing open access publishing of aggregated data through public health agencies or statistics agency websites.

## 4 DISCUSSION

Our analysis used predefined subsystems and criteria to provide a structured comparison of influenza and COVID‐19 surveillance systems in five European countries. This allowed a detailed evaluation of how influenza surveillance was repurposed for COVID‐19, adding to information available from the ECDC^15^ and WHO Regional Office for Europe.^40^ Additionally, there was a dynamic change due to a better understanding of SARS‐CoV‐2 epidemiology, clinical experience and improved diagnostic tools.

Influenza surveillance systems were largely repurposed for COVID‐19 surveillance but shifted from sentinel‐ and syndromic‐based surveillance to systematic and mandatory notification systems that relied heavily on test results. Italy was the first country hit by the pandemic and swiftly implemented centralised and standardised surveillance, highlighting the importance of established influenza surveillance systems as key for pandemic preparedness.^41^ Although the repurposing of existing surveillance tools for influenza was widespread and new components were introduced for COVID‐19, some existing tools such as the monitoring of non‐medically attended events in Germany, Italy and Spain were not reused or were considered inadequate for COVID‐19.

Virological surveillance was repurposed for COVID‐19 to allow regional/national reference laboratories to process suspected cases, and community surveillance was dramatically enhanced to describe the spread of disease through mandatory notification systems complemented by contact tracing strategies. Monitoring COVID‐19 outbreaks in closed settings also improved considerably, covering more settings and with more complete data, especially for care homes.^37^ Primary and secondary care surveillance sentinel schemes were significantly repurposed, and Germany strengthened hospital surveillance by introducing tools covering emergency as well as ICU wards whilst Italy published daily statistics on hospital admissions. In addition, breadth of surveillance increased, with more contributing primary care practices, emergency, hospital and ICU departments. Hospital and care home mortality surveillance systems were re‐used and integrated into national statistics. The EuroMOMO modelling initiative also contributed to measure all‐cause excess mortality.^42^

Improvements in surveillance systems driven by COVID‐19 may have an immediate beneficial impact on influenza surveillance. Broader RT‐PCR use and the introduction of rapid antigen tests in community settings may lead to improvements in diagnostic accuracy. In parallel, the roll‐out of reporting systems across additional medical settings will provide a better picture of the overall burden of respiratory viruses, especially in care homes. Moreover, communication tools and resources developed for COVID‐19 could be re‐used for influenza and other diseases. Improvements such as the evaluation of the pressure on healthcare systems and economic/societal impact could benefit the overall evaluation for influenza. In addition, the degree of adoption of non‐pharmaceutical interventions and vaccine coverage rate will be important considerations in future evaluations. In particular, monitoring laboratory testing capacity and the number of tests performed as a key metric is a direct benefit of the pandemic. Indicators were developed to monitor pressure on healthcare systems, including saturation of hospital capacity, availability of healthcare workers, overall patient attendance and impact on paediatric immunisation. The broader disruption of COVID‐19 on economies and societies was monitored through new indicators, such as GDP or employment rate,^43^ mental health and isolation, school attendance, crime rate or trust in the government.^44^

However, as well as the improvements to influenza surveillance resulting from innovations for COVID‐19, experience in the southern hemisphere warned of an adverse impact of COVID‐19 on influenza surveillance^45^ and our analysis showed similar trends. The similarity in symptoms for COVID‐19 and influenza means that there is a need to better delineate the clinical diagnosis of influenza for syndromic surveillance and patient management systems^3^ although an overreliance on RT‐PCR testing for COVID‐19 diagnosis, regardless of the presence of symptoms, raises doubts over this approach. Importantly, the prioritisation of COVID‐19 and ARI testing can adversely affect surveillance of epidemiologically important viruses (RSV and influenza) for both paediatric and adult populations,^12^ highlighting the need to increase diagnostic resources. Also, a change in health‐seeking behaviours (e.g., teleconsultations) may reduce clinical examinations and virological swabbing in future influenza seasons, which could affect the reliability of results from sentinel schemes.^46^ The intensified surveillance for COVID‐19 has placed increased pressure on healthcare systems throughout Europe, and whilst feasible in the short term for a pandemic situation the routine adoption of similarly intense practices for other disease surveillance is unlikely.

Another effect of the burden of COVID‐19 is the reduction of influenza virological surveillance, which is critical to identify the strains for the next season's vaccine and could have real consequences on the efficacy of future seasonal influenza vaccines as well as pandemic preparedness.^11^, ^12^, ^47^ The WHO has reviewed country experience in repurposing influenza surveillance systems in response to COVID‐19, aiming to develop interim recommendations on how to best sustain influenza sentinel surveillance.^48^ Similarly, the reduced circulation of influenza viruses, largely due to non‐pharmaceutical interventions introduced during the COVID‐19 pandemic, is likely to have consequences for the identification of influenza strains for future vaccines.

Our analysis has some limitations. It is restricted to five western European countries with well‐established influenza surveillance systems, and the results cannot be easily extrapolated to other countries. Also, the focus on national surveillance does not account for regional disparities, which may be considerable in countries with decentralised healthcare systems. The emergence of SARS‐CoV‐2 variants of concern is setting new challenges for surveillance systems and may trigger new adaptations. The capacity of countries to continuously adapt their surveillance to new challenges may also become an indicator for future analysis. Lastly, although the future of influenza surveillance systems is discussed, this analysis does not aim to conclude on the sustainability of current surveillance strategies, as key elements such as cost, human resource or technical feasibility are not considered.

## 5 CONCLUSIONS

Well‐established influenza surveillance systems are a key component of pandemic preparedness and demonstrated efficient repurposing, adaptation and enhancement for COVID‐19 surveillance, complemented by additional components when needed. Although this led to significant improvements in national surveillance systems, which may benefit influenza surveillance, there has also been disruption to existing influenza systems. As COVID‐19 is likely to co‐exist with other respiratory viruses for the upcoming influenza seasons, the challenge will be to find a sustainable path for monitoring all respiratory viruses.

## FUNDING INFORMATION

This work was funded by Sanofi Pasteur.

## AUTHOR CONTRIBUTIONS

**Hélène Bricout:** Conceptualization; data curation; formal analysis; funding acquisition; investigation; methodology; project administration; resources; supervision; validation; visualization. **Thierry Rigoine de Fougerolles:** Conceptualization; data curation; formal analysis; investigation; methodology; project administration; visualization. **Joan Puig‐Barbera:** Conceptualization; formal analysis; methodology; validation. **George Kassianos:** Conceptualization; methodology; validation. **Philippe Vanhems:** Conceptualization; methodology; validation. **Jorg Schelling:** Conceptualization; methodology; validation. **Pascal Crepey:** Conceptualization; methodology; validation. **Raul Ortiz de Lejarazu:** Conceptualization; methodology; validation. **Filippo Ansaldi:** Methodology; validation. **Markus Fruhwein:** Methodology; validation. **Cristina Galli:** Methodology; validation. **Anne Mosnier:** Methodology; validation. **Elena Pariani:** Methodology; validation. **Anvar Rasuli:** Conceptualization; investigation; methodology; project administration; validation. **Olivier Vitoux:** Conceptualization; methodology; project administration; supervision; validation. **John Watkins:** Methodology; validation. **Thomas Weinke:** Methodology; validation.

### PEER REVIEW

The peer review history for this article is available at https://publons.com/publon/10.1111/irv.12941.

## Supporting information


**Data S1.** Influenza and COVID‐19 surveillance system overview

## Data Availability

All data are available in public repositories, referenced throughout the article. Any further requests should be directed to the Corresponding Author.
